# Patient waiting time in the outpatient clinic at a central surgical hospital of Vietnam: Implications for resource allocation

**DOI:** 10.12688/f1000research.11045.3

**Published:** 2017-06-15

**Authors:** Tho Dinh Tran, Uy Van Nguyen, Vuong Minh Nong, Bach Xuan Tran

**Affiliations:** 1 Department of Hepatobiliary Surgery, Viet Duc Hospital, Hanoi City, Vietnam; 2 Outpatient Clinic, Viet Duc Hospital, Hanoi City, Vietnam; 3 Institute for Global Health Innovations, Duy Tan University, Da Nang, Vietnam; 4 Institute for Preventive Medicine and Public Health, Hanoi Medical University, Hanoi City, Vietnam

**Keywords:** Patient waiting time, outpatient clinic, Viet Duc Hospital, health insurance

## Abstract

**Background: **Patient waiting time is considered as a crucial parameter in the assessment of healthcare quality and patients’ satisfaction towards healthcare services. Data concerning this has remained limited in Vietnam. Thus, this study aims to assess patient waiting time in the outpatient clinic in Viet Duc Hospital (Hanoi, Vietnam) in order to enable stakeholders to inform evidence-based interventions to improve the quality of healthcare services.

**Methods: **A cross-sectional study was conducted from June 2014 to June 2015 in the outpatient clinic at Viet Duc Hospital. Waiting time stratified by years (2014 and 2015), months of the year, weekdays, and hours of the day were extracted from Hospital Management software and carefully calculated. Stata 12.0 was employed to analyze data, including the average time (M± SD), frequencies and percentage (%).

**Results: **There was a total of 137,881 patients involved in the study. The average waiting time from registration to preliminary diagnosis in 2014 was 50.41 minutes, and in 2015 was 42.05 minutes. A longer waiting time was recorded in the morning and in those having health insurance.

**Conclusions: **Our results provided evidence that despite the decrease of waiting time from 2014 to 2015, waiting time was much higher among patients having health insurance compared to their counterparts. The findings suggest that human resources promotion and distribution should be emphasized in outpatient clinics and health insurance-related administrative procedures should be simplified.

## Introduction

Patient waiting time is defined as the time patients have to wait before meeting clinical staffs or using health service needed ^1– 3^. Although patient waiting time has been defined as an important indicator in the assessment of healthcare quality ^1^ and patients’ satisfaction towards healthcare services ^2, 3^, lengthy outpatient waiting time has posed a great challenge to maximize healthcare quality ^4^. The patient waiting time varies across settings. In Ireland, a study conducted in an outpatient clinic showed that 50% of patients waited 60% for their appointment ^4^. In Nigeria, 60% patients had to wait 90–180 minutes for receiving examination ^5^. Even in the USA, the average patient waiting time was from 60 minutes in Atlanta to 188 minutes in Michigan ^6^. This issue is worse among countries with low provider-patient ratios ^7^.

Vietnam is among highly populated countries that are fueled by patient overload, especially in the central hospitals ^8^. Thus, extended waiting time has remained highly prevalent. In 2015, a study in Ha Dong General Hospital by Nguyen indicated the average time of medical examination was 96.91 ± 72.16 minutes. The average waiting time was 63.05 ± 62.96 minutes ^9^. In 2012, a study by Le *et al.* conducted in an outpatient clinic suggested that the average time spent from registration to doctors’ conclusions was 246.87 ± 104.55 minutes (4.11 ± 1.7 hours) ^10^. Accordingly, patient waiting time is influenced by various factors, such as working procedure, patient overload and appointment schedule ^11, 12^. Previous study suggests that appropriate operation of medical examinations could shorten patient waiting times ^13^.

Viet Duc is a central hospital, with the aim of ensuring health for Northern Vietnamese patients. The outpatient clinic welcomes hundreds of patients on a daily basis and is often overloaded. Thus, Viet Duc Hospital is always seeking evidence-based solutions to enhance the quality of healthcare services. However, data on patient waiting time in the outpatient clinic at Viet Duc Hospital remains limited. Thus, the aim of this study was to examine patient waiting times in the outpatient clinic, Viet Duc Hospital, thereby enabling the hospital administration to design evidence-based interventions to improve the satisfaction of patients.

## Methods

### Study design and settings

A cross-sectional study was conducted from June 2014 to June 2015 in the outpatient clinic of Viet Duc Hospital (Hanoi, Vietnam). It is the largest surgical center of Vietnam, with approximately 1300 beds and approximately 150,000 patients using outpatient services annually.

### Participants

All patients that underwent a medical examination during this time were eligible for the research. There were no specific exclusion criteria used in this study. Data from a total of 137881 patients were extracted for final analysis.

### Data collection and measurements

Time data was collected via Hospital Management Software, which was developed to support hospital management in Viet Duc Hospital. Data concerning the waiting time for utilizing service was computed as the time that patients met the physicians minus the time that the patient registered. These data were automatically recorded when the patients registered and when they met the physicians. We used secondary data instead of primary data in order to get the accurate data for the analysis. By using time record function from the software, we could identify exactly when the patients used their services needed. Due to using secondary data, we did not collect and report the demographic characteristics of patients.

In this study, variables of interest included health insurance status, the waiting time for health service use, year (2014 and 2015), months of the year, weekdays and hours of the day.

### Statistical analysis

Data was extracted in Microsoft Excel form and Stata 12.0 was employed to analyze data: the average time (M±SD), frequencies and percentage (%). Mann-whitney test was used to test the differences of waiting time among variables. P-value < 0.05 was considered statistical significance. Since we extracted data from the software, there was no bias in this study.

### Ethical approval

The study was approved by the IRB of Viet Duc Hospital, Hanoi, Vietnam. Data collection procedures and the use of data for analysis were also approved by the directors of Viet Duc Hospital. No personal data concerning patients was collected in this study.

## Results

Table 1 illustrates the average waiting time of patients in the outpatient clinic of Viet Duc Hospital. There was a total of 137881 patients who had a medical examination during the time of conducting the research, in which 38298 patients had health insurance, accounting for approximately 27.8%. The average waiting time from registration to preliminary diagnosis in 2014 was 50.41 minutes and in 2015 was 42.05 minutes.

**Table 1.** Patient waiting time over a two-year period grouped by health insurance.

Year	Health insurance			No health insurance			Total		
	N	%	Waiting time (mins) (SD)	N	%	Waiting time (mins) (SD)	N	%	Waiting time (mins) (SD)
**2014**	26174	68.34	52.12 (37.15)	49192	49.40	49.51 (36.26)	75366	54.66	50.41 (36.51)
**2015**	12124	31.66	50.71 (33.97)	50391	50.60	39.96 (33.74)	62515	45.34	42.05 (33.76)
**Total**	38298	100	51.67 (36.66)	99583	100	44.68 (35.19)	137881	100	46.62 (35.50)
**p-value**	>0.05								

Patient waiting time regarding the hours of the day are presented in Table 2. The largest number of patients having a medical examination were in the hours 7:00–8:00 and 8:00–9:00. The lowest number of patients having medical examination were in the hours 11:00–12:00, 15:00–16:00 and 16:00–16:30 (because the hospital was closed at 16:30). The longest patient waiting time was at 6:30 to 7:00, and the time among those having health insurance was 81.54 minutes, while the longest patient waiting time among those who did not have health insurance was 70.63 minutes.

**Table 2.** Patient waiting time regarding hours of the day grouped by health insurance.

Time	Health insurance			No health insurance			Total			p-value
	N	%	Waiting time (mins)	N	%	Waiting time (mins)	N	%	Waiting time (mins)
**6:30–7:00**	3507	9.16	81.54 (35.79)	13704	13.76	70.63 (35.85)	17211	12.48	72.85 (35.93)	<0.05
**7:00–8:00**	11498	30.02	60.95 (38.17)	26512	26.62	55.01 (36.98)	38010	27.57	56.80 (37.25)	>0.05
**8:00–9:00**	10631	27.76	47.34 (34.74)	24506	24.61	38.48 (33.54)	35137	25.48	41.16 (33.84)	>0.05
**9:00–10:00**	5238	13.68	40.84 (27.96)	15165	15.23	32.85 (26.33)	20403	14.80	34.90 (26.63)	<0.05
**10:00–11:00**	2339	6.11	39.08 (26.55)	5891	5.92	30.06 (27.25)	8230	5.97	32.62 (27.11)	>0.05
**11:00–12:00**	727	1.90	74.87 (65.77)	1418	1.42	60.21 (54.36)	2145	1.56	65.18 (57.64)	<0.05
**13:00–14:00**	2177	5.68	34.32 (27.32)	6140	6.17	27.73 (28.48)	8317	6.03	29.45 (28.33)	>0.05
**14:00–15:00**	1336	3.49	26.26 (24.83)	4304	4.32	20.56 (24.84)	5640	4.09	21.91 (24.88)	>0.05
**15:00–16:00**	774	2.02	20.91 (27.39)	1743	1.75	24.71 (25.67)	2517	1.83	23.54 (26.01)	>0.05
**16:00–16:30**	71	0.19	45.51 (23.60)	200	0.20	87.48 (45.45)	271	0.20	76.48 (44.49)	<0.05

Table 3 shows patient waiting time regarding weekdays. The largest number of patients having a medical examination was on Monday, Tuesday and Wednesday. There were fewer patients on Thursday and Friday. The shortest waiting time was on Thursday, while the longest waiting time was on Tuesday.

**Table 3.** Patient waiting time regarding weekdays grouped by health insurance.

Weekdays	Health insurance			No health insurance			Total			p-value
	N	%	Waiting time (mins)	N	%	Waiting time (mins)	N	%	Waiting time (mins)	
**Monday**	8655	22.60	52.38 (44.71)	23484	23.58	46.50 (43.67)	32139	23.31	48.08 (44.38)	>0.05
**Tuesday**	9647	25.19	55.00 (38.01)	22931	23.03	46.91 (35.64)	32578	23.63	49.30 (36.12)	<0.05
**Wednesday**	8123	21.21	48.89 (36.69)	19302	19.38	43.25 (35.72)	27425	19.89	44.92 (35.92)	>0.05
**Thursday**	6440	16.82	47.98 (35.75)	16746	16.82	42.21 (34.50)	23186	16.82	43.81 (34.77)	>0.05
**Friday**	5433	14.19	53.18 (35.55)	17120	17.19	43.22 (33.88)	22553	16.36	45.62 (34.24)	<0.05

Table 4 demonstrates patient waiting time regarding the month of the year. Generally, few patients had medical examinations in February, 2015. The longest waiting time was in July, August, and September for both insured and uninsured patients.

Raw data used in the construction of Table 1–Table 4 Data from June 2014–June 2015 detailing waiting times of patients and if health insurance was present. Click here for additional data file. Copyright: © 2017 Tran TD et al. 2017 Data associated with the article are available under the terms of the Creative Commons Zero "No rights reserved" data waiver (CC0 1.0 Public domain dedication).

**Table 4.** Patient waiting time by month of the year grouped by health insurance.

Year	Month	Having health insurance			No health insurance			Total			p-value
		N	%	Waiting time (mins)	N	%	Waiting time (mins)	N	%	Waiting time (mins)
**2014**	**6**	3585	13.70	52.45 (38.24)	6966	14.16	51.69 (36.41)	10551	14.00	51.95 (37.05)	>0.05
	**7**	4212	16.09	59.23 (37.83)	8124	16.51	58.77 (37.34)	12336	16.37	58.93 (37.34)	>0.05
	**8**	3417	13.05	57.79 (37.55)	6925	14.08	58.20 (37.52)	10342	13.72	58.06 (37.54)	>0.05
	**9**	3524	13.46	57.93 (37.42)	6901	14.03	56.55 (33.71)	10425	13.83	57.02 (37.95)	>0.05
	**10**	4078	15.58	53.50 (37.49)	7541	15.33	47.41 (35.89)	11619	15.42	49.55 (36.47)	>0.05
	**11**	3667	14.01	42.19 (33.60)	6200	12.60	36.90 (33.34)	9867	13.09	38.87 (33.46)	>0.05
	**12**	3691	14.10	41.23 (36.15)	6535	13.28	33.38 (30.23)	10226	13.57	36.21 (30.23)	>0.05
**2015**	**1**	2042	16.84	47.47 (34.28)	7747	15.37	33.02 (31.43)	9789	15.66	36.04 (32.14)	<0.05
	**2**	796	6.57	42.23 (35.19)	4574	9.08	33.93 (31.32)	5370	8.59	35.16 (31.32)	<0.05
	**3**	2343	19.33	50.02 (39.22)	9986	19.82	37.72 (34.83)	12329	19.72	40.06 (34.83)	<0.05
	**4**	2082	17.17	49.79 (34.63)	8557	16.98	39.93 (31.90)	10639	17.02	41.86 (32.30)	<0.05
	**5**	2387	19.69	52.22 (40.12)	9271	18.40	43.40 (34.66)	11658	18.65	45.21 (34.66)	<0.05
	**6**	2474	20.41	56.06 (36.17)	10256	20.35	47.00 (35.00)	12730	20.36	48.76 (34.98)	<0.05

## Discussion

The purpose of this study was to assess the patient waiting time in an outpatient clinic, Viet Duc Hospital, Hanoi, Vietnam. Our findings indicate that the average waiting time from registration to preliminary diagnosis was decreased in a period of two years from 2014 to 2015. Findings also suggest the difference regarding waiting time between the morning and the afternoon, those having health insurance compared to those that did not have health insurance.

The average waiting time was lower than previous studies at Ha Dong General Hospital (Hanoi City) ^9^, Trung Vuong Emergency Hospital (Ho Chi Minh city) ^10^, and Nguyen Trai Hospital (Ho Chi Minh City) ^14^. However, our findings were higher than studies by Vu at the National Hospital of Tropical Diseases (Hanoi City) ^13^, and Cole in Australia ^15^. It could be hypothesized that the outpatient clinic at Viet Duc Hospital is well-qualified (with skilled physicians and advanced medical technologies), patients directly come to the Hospital without visiting healthcare facilities at grass-roots levels, leading to overload. In fact, each department at the hospital receives approximately 130,000 medical visits every year; therefore, overload frequently happens. The study in Trung Vuong Emergency Hospital was conducted in 2011 when the decision 1313/QĐ-BYT related to the medical examination procedure was not implemented. Therefore, patient waiting time might be prolonged.

The higher number of visited patients in the morning and the afternoon observed in our study could be potentially explained since patients prefer to have health consultations in the morning, as they could receive the results of clinical tests within the day. A study by Han *et al.* also indicated that the number of patients that visit An Giang Cardiovascular Hospital (An Giang Province) in the morning is higher than the afternoon ^16^. Thus, our study highlights the essential need for well-distribution of human resources to shorten patients’ time of medical consultations.

Noticeably, those having health insurance had to wait for their turn longer than those that did not have health insurance. This may potentially reflect shortcomings regarding complicated administrative procedures that could extend waiting time ^9^. In fact, cumbersome administrative procedures related to health insurance remain the pressing issue in Vietnamese healthcare system ^17^. Since this strategy may be hampered by health insurance-related procedures, stakeholders should pay attention on simplifying administrative procedures for insured patients.

## Conclusions

Our results provided evidence that despite the decrease of waiting time from 2014 to 2015, waiting time was much higher among patients having health insurance compared to their counterparts. The findings suggest that human resources promotion and distribution should be emphasized in outpatient clinics and health insurance-related administrative procedures should be simplified.

## Data availability

The data referenced by this article are under copyright with the following copyright statement: Copyright: © 2017 Tran TD et al.

Data associated with the article are available under the terms of the Creative Commons Zero "No rights reserved" data waiver (CC0 1.0 Public domain dedication). 

**Dataset 1: Raw data used in the construction of Table 1– Table 4.** Data from June 2014-June 2015 detailing waiting times of patients and if health insurance was present. doi, 10.5256/f1000research.11045.d157112 ^18^
