# Supplementary material for: Patient waiting time in the outpatient clinic at a central surgical hospital of Vietnam: Implications for resource allocation
Source: F1000Res. 2017 Jun 15;6:454. Originally published 2017 Apr 10. [Version 3] doi: 10.12688/f1000research.11045.3 (PMC5482327; doi:10.12688/f1000research.11045.3)
Supplement: Peer review history

# Peer review: Referee response for version 3

**DOI:** 10.5256/f1000research.12864.r23493

**Published:** 2017-06-16

**Authors:** Ozayr H. Mahomed

**Affiliations:** 1 Public Health Medicine, University of KwaZulu-Natal, Durban, South Africa

Introduction- "The patient waiting time varies across settings. In Ireland, a study conducted in an outpatient clinic showed that 50% of patients waited 60% for their appointment"

 What does 60% mean or is this an error that required minutes?

 Results: The average waiting time from registration to preliminary diagnosis in 2014 was 50.41 minutes and in 2015 was 42.05 minutes- Please write the SD in the text

 In table 2 you have p values calculated but there is no mention of this in the text when describing the waiting times in each time category. Were they significant or not?

 Your tables are not presented in the conventional manner. We do not place p values in tables. We highlight significance by * for <0,05 and ** p=<0,01 and have the notion at the bottom.

I have read this submission. I believe that I have an appropriate level of expertise to confirm that it is of an acceptable scientific standard.

# Peer review: Referee response for version 2

**DOI:** 10.5256/f1000research.12575.r22865

**Published:** 2017-05-22

**Authors:** Ozayr H. Mahomed

**Affiliations:** 1 Public Health Medicine, University of KwaZulu-Natal, Durban, South Africa

Thank you for providing me the opportunity to review the article. An important topic indeed. However, I think that the introduction is very superficial without adequately contextualising the waiting time problem.

 Methodology is very superficial indeed. There is no indication of what additional parameters were added to the hospital management system. What parameters were used to obtain the data? Who enters the data? When is patient waiting time measured from?

 Why did the authors do a secondary data analysis and did not conduct primary data collection?

 Results - There is no general profile of the patients provided. There is no standard deviation or confidence intervals provided. The results could be better presented using histograms for times of the day.

 Discussion - This is highly superficial. There is no mention of queuing theory or application thereof. The study does not provide any human resource data and therefore cannot link the discussion on human resources requirements. The findings were not disaggregated by health insurance and therefore the last paragraph of the discussion cannot be linked to relevant data.

 Conclusion - Are tailored to suit the authors agenda but not based on the findings of the study or the aims.

I have read this submission. I believe that I have an appropriate level of expertise to confirm that it is of an acceptable scientific standard, however I have significant reservations, as outlined above.

# Response

**Published:** 2017-06-10

**Authors:** Vuong Nong Minh

**Affiliations:** Institute for Global Health Innovations, Duy Tan University, Vietnam

Dear Dr.Mahomed,

Thank you very much for your comments. We have carefully clarified and added more details in the updated manuscript. We believe that this version is sufficient for your acceptance.

Sincerely,

Minh Vuong and co-authors

---------------------------------------------

Comments:

1) However, I think that the introduction is very superficial without adequately contextualising the waiting time problem.

Response: We have added more literatures in this section

2) Methodology is very superficial indeed. There is no indication of what additional parameters were added to the hospital management system. What parameters were used to obtain the data? Who enters the data? When is patient waiting time measured from? Why did the authors do a secondary data analysis and did not conduct primary data collection?

Response: We have added more details and clarified why we used secondary data in this section.

3) Results - There is no general profile of the patients provided. There is no standard deviation or confidence intervals provided. The results could be better presented using histograms for times of the day.

Response: We have added standard deviation in each table.

4) Discussion - This is highly superficial. There is no mention of queuing theory or application thereof. The study does not provide any human resource data and therefore cannot link the discussion on human resources requirements. The findings were not disaggregated by health insurance and therefore the last paragraph of the discussion cannot be linked to relevant data.

Response: We have discussed more the result. The findings have been disaggregate by health insurance, therefore we believe that we can draw the implications as in the discussion.

5) Conclusion - Are tailored to suit the authors agenda but not based on the findings of the study or the aims. 

Response: We have rewritten the conclusion in order to be appropriate with the result.

# Peer review: Referee response for version 2

**DOI:** 10.5256/f1000research.12575.r22715

**Published:** 2017-05-16

**Authors:** Duong Minh Duc

**Affiliations:** 1 Department of Reproductive Health, Hanoi University of Public Health, Hanoi, Vietnam

Thanks for the revision. The paper has adapted to my previous comments.

I have read this submission. I believe that I have an appropriate level of expertise to confirm that it is of an acceptable scientific standard.

# Peer review: Referee response for version 1

**DOI:** 10.5256/f1000research.11913.r21992

**Published:** 2017-05-10

**Authors:** Duong Minh Duc

**Affiliations:** 1 Department of Reproductive Health, Hanoi University of Public Health, Hanoi, Vietnam

The paper is short and concise but the statistical test is too simple. The authors should use some bivariate analysis. 

 The discussion about "Moreover, the government has planned to move toward universal health insurance, where 80% of the total population are covered by health insurance and reduce out-of-pocket health expenses to under 40% by 2020. Since this strategy may be hampered by health insurance-related procedures, stakeholders should pay attention on simplifying administrative procedures for insured patients." could be not appropriate because this study has been conducted in a national-level hospital and it could not be refer to universal health coverage which should be provided at grassroot level (commune health station or district hospital).

I have read this submission. I believe that I have an appropriate level of expertise to confirm that it is of an acceptable scientific standard, however I have significant reservations, as outlined above.

# Response

**Published:** 2017-05-11

**Authors:** Vuong Nong Minh

**Affiliations:** Institute for Global Health Innovations, Duy Tan University, Vietnam

Dear Mr Duc,

Thank you very much for your comments. We would very carefully consider your feedback and revise our manuscript. We hope that our newest version makes you satisfy.

Sincerely,

Authors
